# Creating semiconductor metafilms with designer absorption spectra

**DOI:** 10.1038/ncomms8591

**Published:** 2015-07-17

**Authors:** Soo Jin Kim, Pengyu Fan, Ju-Hyung Kang, Mark L. Brongersma

**Affiliations:** 1 Geballe Laboratory for Advanced Materials, 476 Lomita Mall, Stanford, California 94305-4045, USA.

## Abstract

The optical properties of semiconductors are typically considered intrinsic and fixed. Here we leverage the rapid developments in the field of optical metamaterials to create ultrathin semiconductor metafilms with designer absorption spectra. We show how such metafilms can be constructed by placing one or more types of high-index semiconductor antennas into a dense array with subwavelength spacings. It is argued that the large absorption cross-section of semiconductor antennas and their weak near-field coupling open a unique opportunity to create strongly absorbing metafilms whose spectral absorption properties directly reflect those of the individual antennas. Using experiments and simulations, we demonstrate that near-unity absorption at one or more target wavelengths of interest can be achieved in a sub-50-nm-thick metafilm using judiciously sized and spaced Ge nanobeams. The ability to create semiconductor metafilms with custom absorption spectra opens up new design strategies for planar optoelectronic devices and solar cells.

## Abstract (web summary)

Ultrathin semiconductor metafilms can be designed to achieve near-unity absorption in specific spectral regions. Here, Kim *et al*. engineer nanoscale optical resonances in sub-50-nm-thick germanium nanobeams metafilms to demonstrate near-unity absorption in one or more desired wavelength regions.

## Graphical abstract

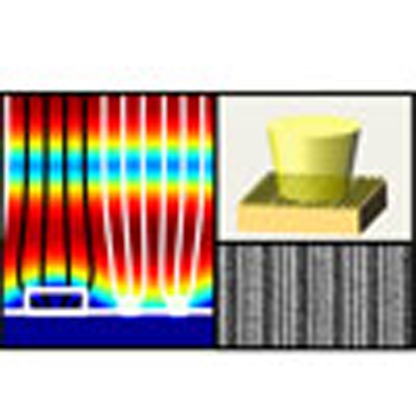

The achievement of strong light absorption in ultrathin layers of material has been of great scientific and practical interest for many years. The early works by Woltersdorf^1^ and Salisbury^2^,^3^ pointed to the intriguing possibility of achieving near-unity optical absorption in a deep-subwavelength layer of material. The realization of such ultrathin absorber layers can increase performance and reduce fabrication and materials cost of many thin-film light absorber applications, such as solar cells^4^,^5^, photoelectrochemical water splitting^6^,^7^,^8^, photodetection^9^,^10^ and thermal engineering applications, such as thermal photovoltaics and imaging^11^,^12^. Thinner layers naturally also come with an increased mechanical flexibility that is desired in a variety of device technologies^13^. Unfortunately, the intrinsic optical properties of materials found in nature are fixed, and thus offer little freedom in spectrally controlling and enhancing absorption. In contrast, metamaterials constructed from subwavelength arrays of metallic^14^,^15^,^16^,^17^ or semiconductor^18^,^19^ structures afford significant flexibility in manipulating the effective optical materials properties and this presents a tantalizing opportunity to realize designer super-absorbers with atomic-scale control^20^,^21^,^22^,^23^,^24^,^25^.

Here we build on recent developments in our understanding of the resonantly enhanced light absorption properties of individual high-refractive-index semiconductor nanostructures^18^,^19^,^26^,^27^ to create broadband, super-absorbing semiconductor metamaterial layers or ‘metafilms' that are <50 nm in thickness. We start by analysing the light absorption properties of a metafilm constructed from a single type of resonant building block, a Ge nanobeam, placed on a metal back reflector. We demonstrate that near-unity absorption can be achieved at different target operation wavelengths that are identical to the peak absorption frequency of the individual beams. This implies that the optical interactions between very closely spaced, high-index building blocks are very weak. At first sight, such behaviour may not be anticipated for deep-subwavelength dielectric nanostructures whose modal fields extend well beyond their physical boundary, and thus probe their environment. Moreover, even plasmonic nanostructures that are known for their extreme field confinement frequently show strong optical coupling between closely spaced particles^28^ as well as in metamaterials where spectral splitting results from strong coupling between vertically stacked or laterally spaced metal nanostructures^29^,^30^. The difference in coupling behaviour results from the fact that high-index particles tend to have the dominant fraction of the field internal to the structure and metallic particles tend to push fields out to the exterior^31^.

Here we show that the weak coupling in semiconductor systems can effectively be exploited to realize multiresonant semiconductor metafilms in which differently sized resonant building blocks are placed at deep-subwavelength spacings to achieve broadband, near-unity absorption. The possibility to pack resonant semiconductor nanostructures with large absorption cross-sections at spacings well below the diffraction limit (*d*<<*λ*/2) suggests an intriguing new opportunity to create (meta)films with very high optical mode densities, which is desired for reaching the fundamental limits to solar light trapping^32^,^33^,^34^,^35^,^36^. Whereas for plasmonic nanostructures this type of approach has been proven successful to achieve more broadband absorption^24^,^25^, it has not been explored to what extent and how the absorption spectrum can be engineered with semiconductor nanostructures.

## Results

### Realizing metafilms with designer absorption spectra

**Figure 1:**
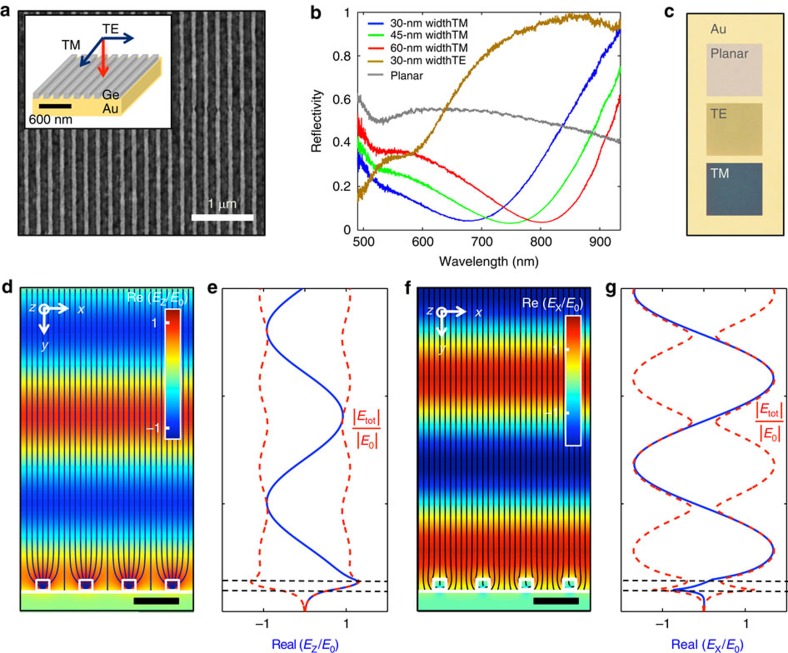
Optical properties of a semiconductor metafilm constructed from an array of resonant Ge nanobeams placed on a metallic back reflector. (**a**) Top-view scanning electron microscopy image of a fabricated metafilm with a 200-nm-period and 0.3-duty cycle Ge beams. Scale bar, 1 μm. (**b**) Measured reflectivity spectra for TM polarized light reflecting from metafilms constructed from nanobeams 30 (blue), 45 (green) and 60 nm beams (red). The reflectivity spectra for TE polarized light reflecting of the array with 30-nm-wide beams (brown) and a planar Ge film (grey) are shown for reference. (**c**) Optical images of the planar Ge film (top), and the nanobeam arrays with 30-nm-sized beams under TE (middle) and TM illumination (bottom). The appearance of the Au film is shown in the background. (**d**) Electric field distribution and power flow for a TM polarized plane wave at *λ*=800 nm that is normally incident on a metafilm with 60-nm-wide beams. The cross-section of the beams (oriented in the *z* direction) and substrate are outlined in white. The magnitude of the field is normalized to the incident field (*E*_0_). Scale bar, 200 nm. (**e**) Normalized electric field amplitude (Re(*E*_*z*_/*E*_0_) and |*E*_tot_/*E*_0_|) along a vertical cross-section that passes through the centre of a nanobeam. (**f**,**g**) Same analysis for TE polarization as in **d** and **e**.

Figure 1a shows a scanning electron microscopy image of a periodic array of 48-nm-thick and 60-nm-wide Ge nanobeams placed on a 150 nm thickness of gold (Au)-coated substrate. This pattern was generated using standard electron-beam lithography, thin-film deposition and etching techniques (see Methods). Figure 1b shows the normal incidence reflection spectra taken for transverse magnetic (TM) polarized light (red curve) with the electric field parallel to the length of the beams and for the orthogonal transverse electric (TE) polarization (brown curve). For TM polarization, a strong dip in the reflectivity is observed at a wavelength of 810 nm. This reflection minimum can continuously be blue-shifted by >100 nm in wavelength when the width of the beams *w* is reduced to 45 nm (green curve) and ultimately 30 nm (blue curve) while keeping the Ge filling fraction *f*_Ge_ constant at 0.3. The optical behaviour is very different for TE polarized light and for randomly polarized light reflecting from a reference sample with an unpatterned 48-nm-thick Ge film on Au (grey curve). These samples show substantially higher reflectivities on the long-wavelength side of the spectrum. The difference in optical behaviour can also be observed directly in bright-field optical reflection images taken from the nanobeam samples under white light illumination (Fig. 1c). Below we will argue that the wavelength-tunable dip in the TM reflection spectra results from strong light absorption induced by the excitation of an optical resonances supported by the nanobeams.

It is important to note that the observed tunability of the reflection dip is not predicted by a first-order effective medium theory. The effective permittivity for TM polarized light according to this theory is: *ɛ*_eff_*=f*_Ge_ *ɛ*_Ge_*+*(1*-f*_Ge_)*ɛ*_air_ (refs 37, 38), where *ɛ*_Ge_ and *ɛ*_air_ are the permittivities of Ge and air, respectively. Within this approximation, the optical properties only depend on the materials fractions and not the geometry. For this reason, no changes in the optical properties are expected given the fixed *f*_Ge_=0.3. This zeroth-order approximation can accurately predict the behaviour of a nanobeam array in the limit that the width and period are deep subwavelength (*w*, *P* <<*λ*) and the normalized thickness *t*/*λ*>>1 (ref. 39). In that limit, the high diffraction orders are evanescent and decay. As such, only the zeroth order can propagate and is relevant to the optical properties of the array. The first-order theory does make for an interesting reference case as it does not capture the impact of optical resonances capable of redistributing the fields from an incident wave to resemble those of the resonant modes supported by the nanobeams. A comparison to full-field simulations (which do naturally take into account resonances) enables study of the impact of resonances on the spectral absorption properties.

Next, we use full-field electromagnetic simulations to show that an optical resonance supported by the individual nanobeams facilitates the strong, tunable absorption for TM polarized light seen in Fig. 1b,c. The optical power flow and field analysis in Fig. 1d,e illustrate how a normally incident, TM polarized light wave at 800-nm wavelength is funnelled into the 60-nm-wide resonant Ge beams of a metafilm. Such a redirection of light is commonly seen in the near-field of optically resonant nanostructures and attributed to magnetoelectric interference^40^. In this case, the high-index nanobeams support optical Mie-like^41^ resonances that are associated with the excitation of the leaky optical modes of such nanostructures^26^,^42^. The resonances in the Ge beams are sufficiently strong to capture virtually all of the incident light with hardly any of the power flow lines hitting/ending up in the metal film. Unity absorption is reached when the (linear) absorption cross-section matches the nanobeam period. This is possible as the absorption cross-section *σ*_Abs_ of resonant structures can exceed their geometrical cross-section *σ*_Geom_, that is, an absorption efficiency *Q*_Abs_=*σ*_Abs_/*σ*_Geom_ >1 (ref. 41). The funnelling action explains the very strong light absorption in the Ge nanobeams and the deep reflection dip in Fig. 1b. The small reflectivity on resonance results in a very weak oscillation of the electric field above the metal film (Fig. 1e). Due to the strong intrinsic materials absorption of Ge, a low-quality factor optical resonance and an associated small field enhancement in the nanobeams over the incident field suffices to achieve near-unity absorption. Strong absorption in this case is simply attained by achieving a natural flow of the incident light into the beams without a reflection, that is, achieving a good impedance match. The low-quality factor resonance ensures that the strong absorption is spectrally broad.

For TE polarization, there are no resonant modes supported around 800 nm for these very small beams. As a result, the power flow is only weakly perturbed from the incident power flow and the strong oscillation in the field distribution above the metafilm is indicative of a strong reflection from the Au substrate (Fig. 1f). The field maximums of the standing wave profile are displaced from those for TM polarization. The first maximum is observed about a quarter wavelength above the metal surface, similar to the case of a good metallic reflector. Since the effective index of the metafilm for the TE polarization is quite low across the considered wavelength range (for example, *n*_eff_=1.7+*i*0.4 at 800 nm as obtained from an effective parameter extraction procedure^43^), the strongest reflection occurs at the Au substrate rather than the top of the metafilm. This explains the Au-like appearance for TE polarized illumination in Fig. 1c. The zero crossing seen in the vertical field profile (Fig. 1g) within the metafilm and the local maximum at the metal surface is linked to the excitation of surface plasmon polaritons by the nanobeams. The poor overlap of the field with the beams leads to relatively weak absorption.

**Figure 2:**
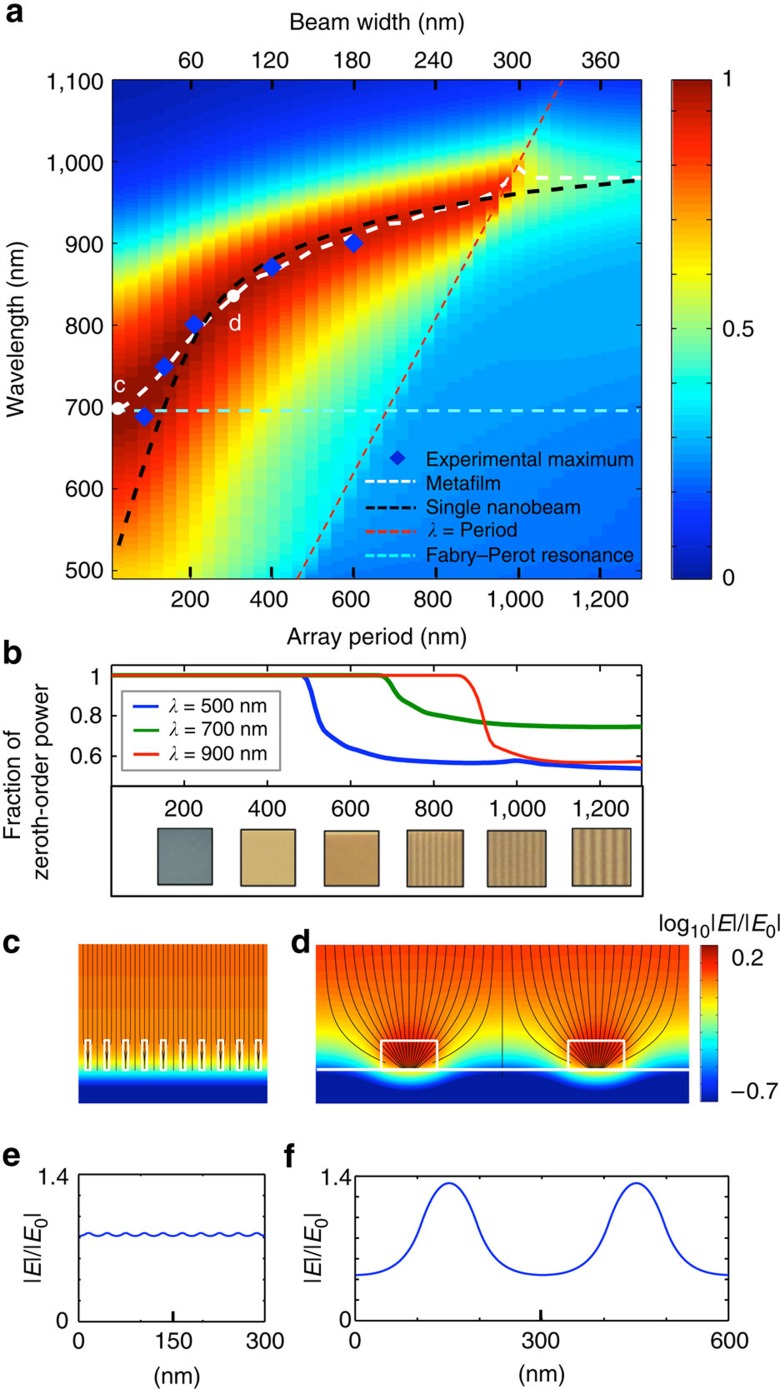
Link between the absorption properties of a metafilm and the constituent nanobeams. (**a**) Simulated absorption map for Ge metafilms with different nanobeam periods (P)/widths (*w*) at a constant filling fraction of *f*_Ge_=0.3 under TM illumination. The wavelength of maximum absorption for the metafilm (white dashed line) closely tracks the wavelength of maximum absorption for single nanobeams (black dashed line) over a broad range of periodicities/widths. At sub-200-nm periodicities, the resonant properties of the metafilm deviate from those of the single beams. At sub-30-nm periodicities, the effective optical properties of the metafilm approach the first-order effective medium theory and the character of the metafilm resonance properties changes from Mie-like to a thin-film, Fabry–Perot style resonance. The spectral location of the Fabry–Perot resonance predicted by the first-order effective medium theory is given by the horizontal cyan dashed line. For reference, the red dashed line denoted the points where *λ*=*P*. The blue dots are the experimentally measured maximum absorption wavelengths. (**b**) Simulated fraction of the zeroth-order reflected power versus the array period for wavelengths of 500 (blue), 700 (green) and 900 nm (red). White light reflection images of fabricated samples featuring different periodicities. (**c**–**f**) Electric field distributions (**c**,**d**) and amplitude of electric field along the lateral direction and through the centre of the beams (**e**,**f**) for two representative examples of metafilms in which the beams do not support a resonance (point c in **a**) and do support a resonance (point d in **a**). The magnitude of the field is normalized to the incident field.

To quantify and better understand how the resonant optical properties of the beam-array emerge from those of the individual nanobeams, we map the absorption for TM polarized light versus the wavelength and array period (Fig. 2a). We again keep the Ge filling fraction constant at *f*_Ge_=0.3 and an increase in the periodicity, thus, implies an increase in the beam width. The TE case and the control over periodicity with fixed beam width are analysed in Supplementary Fig. 1 and Supplementary Fig. 2, respectively. The absorption in the Ge beam-array (excluding metal absorption) is calculated using full-field simulations. The red band shows that near-unity absorption can be achieved for all array periods below 950 nm. This strong absorption occurs in the map above the dashed red line at which the wavelength equals the array period (*λ*=*P*). This is the regime where no diffracted orders are generated in reflection and a description of the beam-array as a metafilm is of practical value. Below the red line, diffracted beams can carry away optical power and the light absorption is relatively weak (<50%). Figure 2b shows the fraction of the power carried by the zeroth diffracted order for the wavelengths of 900 (red line), 700 (green line) and 500 nm (blue line). The occurrence of higher diffracted orders allows the nanobeams to be resolved in optical images (Fig. 2b), making a description of the beam-array as a metafilm inappropriate.

The dashed white line, which highlights the points of maximum absorption in the map, indicates that the absorption peak red-shifts from ∼700 to 950 nm with increasing period. The peak absorption over a wide range of periods (200–950 nm) closely tracks the simulated peak absorption of the individual beams (dashed black line), which features a red-shift with increasing beamwidth^26^,^42^. Below a 200-nm period, the resonant properties of the individual beams and metafilm diverge. For these periods, the dashed white line deviates from the dashed black line and instead approaches the dashed cyan line. The cyan line shows the wavelength for which the first-order metamaterial theory would predict the occurrence of a thin-film Fabry–Perot resonance. The electric field distributions for arrays with periodicities of 30 and 300 nm (Fig. 2c) provide insight into the change in behaviour with decreasing period. For the 300-nm-period array, there are significant variations in the lateral electric field distribution. There is one strong anti-node in the field at the centre of the beams, indicative of the excitation of the lowest-order nanobeam resonance. For the 30-nm-period array, only small spatial variations in the field amplitude are seen. Here the small size and proximity of beams causes the resonant behaviour in the beams to disappear. As such, the effective optical properties of the metafilm can be captured by the first-order effective medium theory and character of the resonant behaviour changes from Mie-like resonance in the beams to a thin-film Fabry–Perot style resonance. The blue diamonds indicate experimentally determined values of the minimum in reflection (that is, maximum absorption) for five different array periods. Good agreement between theory and experiment is obtained, indicating that there is a large range of beam periods/sizes for which the resonant optical properties of the individual nanobeams are preserved in the optical properties of the metafilm. Also, the experimentally measured reflection <5% (Fig. 1b) agrees well with the theoretically predicted absorption (>95%) at each target wavelength.

### The creation of broadband, multiresonant metafilms

When the goal is to achieve strong light absorption over a broad spectral range, it is of value to consider light trapping theory developed for solar cells. Such theories have established fundamental limits to light trapping through an analysis of the relevant electromagnetic modes a cell^32^,^33^,^34^,^35^,^36^. For conventional cells constructed from planar semiconductor layers, grating-like structures couple sunlight with guided-mode resonances by which the light spends some time propagating in the semiconductor layer. With the aim to save on materials and synthesis costs, there have been great strives to reduce the cell thickness while maintaining a high power conversion efficiency. As a result, solar cells operate over an increasing bandwidth in the weak absorption limit where the product of the absorption coefficient *α* and the cell thickness obeys: *αt*<<1. In this regime, it is of value to achieve overcoupling with the system's resonances to maximize the absorption enhancement derived from light trapping^35^. By the nature of overcoupling, some photons are necessarily unused. In the proposed metafilms, the relevant modes are the leaky, resonant optical modes of the nanobeams, which are very different in character. For this reason, it is of interest to explore the possibility to achieve critical coupling over a large bandwidth with such structures. The reflection data in Fig. 1b show that using very thin Ge nanobeams can feature absorption cross-sections that significantly exceed their geometric cross-sections. As a result, one can operate in the critical-coupling regime for which unity absorption is reached by the metafilm. Whereas it is of great value to achieve such strong absorption, the bandwidth is limited (>90% absorption over about 100 nm). Next, we explore the possibility to increase the bandwidth using semiconductor nanostructure with distinct resonance frequencies and spacing them at distances that are small compared with the diffraction limit.

**Figure 3:**
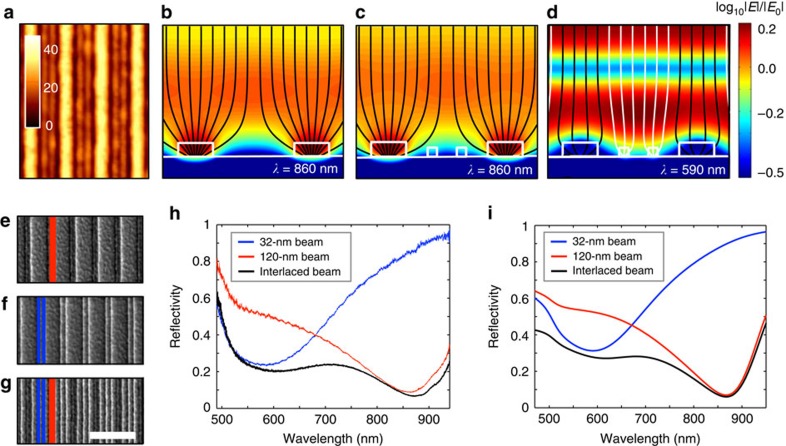
Design and optical properties of a multiresonant metafilm. (**a**) Atomic force microscopy image of the metafilm constructed from two differently sized Ge nanobeams of 120 and 32 nm. Scale bar shows the height of the structures in nanometres. (**b**–**d**) Electric field distributions with power flow lines for the metafilm with only the 120-nm-wide beams spaced by 400 nm (**b**) and the multiresonant metafilm with both 120-nm-wide and 32-nm-wide beams illuminated at wavelengths of 860 (**c**) and 590 nm (**d**). The magnitude of the field is normalized to the incident field. (**e**–**g**) Scanning electron microscopy (SEM) images of the metafilm with only 120-nm-wide beams (**e**), with only narrow 32-nm beams (**f**) and with interlaced narrow and wide beams (**g**). Scale bar, 800 nm (for the SEM images) (**h**,**i**). Experimental reflectivity spectra *R*_metasurface_ providing an estimate for the absorption of the metafilm (**h**) and the simulated reflectivity spectrum (**i**).

Figures 1 and 2 showed that near-unity absorption can be achieved in metafilms that feature resonant nanobeams that are significantly smaller than their interspacing. This leaves significant amounts of room between neighbouring beams for placement of other, differently sized beams that feature distinct resonance frequencies. To achieve more broadband absorption, it is desirable to choose nanobeams that feature closely spaced, but spectrally non-overlapping resonances. Figure 3a shows an atomic force microscopy image of an optimized metafilm that achieves this goal by interlacing Ge nanobeams with widths of 120 and 32 nm. On the basis of Fig. 2a, a metafilm with just the 120-nm-wide beams spaced by 400 nm can exhibit near-unity absorption at 860 nm. This is confirmed by a plot of the power flow that shows virtually all of the light at this wavelength can be collected by the nanobeams (Fig. 3b). When two smaller 32-nm-wide beams are interlaced, the power flow at this wavelength is hardly affected (Fig. 3c). However, the presence of the smaller beams significantly enhances the light absorption at their resonance wavelength of 590 nm. At this wavelength, the large beams are not on resonance and their absorption efficiency is reduced to *Q*_Abs_=0.9. However, the reduced absorption by the large beams is compensated by the small beams that feature a *Q*_Abs_ as large as 2.9 on resonance.

To experimentally verify that the absorption spectrum of a multiresonant metafilm can be designed with knowledge of the resonant properties of the individual building blocks, we consider three representative samples shown in Fig. 3e,f,g. These figures show scanning electron microscopy images of metafilms with just 32-nm-wide beams (Fig. 3e), just 120-nm-wide beams (Fig. 3f) and a sample with these beams interlaced (Fig. 3g). Spectral reflectivity measurements for the metafilms under TM illumination are shown in Fig. 3h. The red, blue and black lines, respectively, show the reflectivities for the samples with just large beams, just small beams and the interlaced array. From these spectra, it is clear that the samples with a single-beam size show a single reflection dip near the long/short resonance wavelength that is characteristic for the wide/narrow individual beams. The interlaced array shows two reflection dips at the exact spectral locations where the individual beams are resonant. It is clear that the overall, broadband absorption is enhanced for the interlaced sample over each of the samples with one type of beam. These measurements are in good agreement with the theoretical simulations shown Fig. 3i. Minor differences are possibly due to the sample-to-sample variations in the measured refractive index of a-Ge that were used in the simulations as well as small discrepancies in the simulated shape and size from those in the experiments.

**Figure 4:**
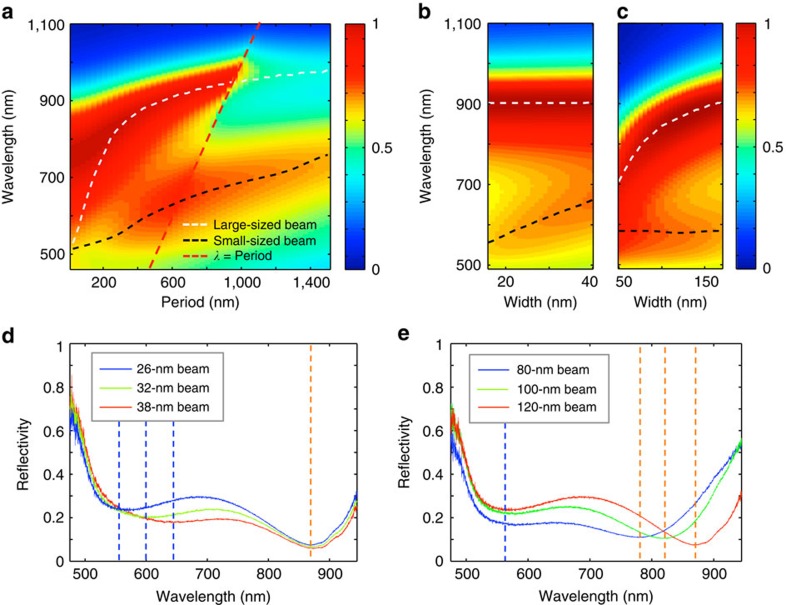
Creating designer absorption spectra of a metafilm by tuning the resonances of the constituent nanobeams. (**a**) Absorption map of a multiresonant metafilm under TM illumination. The filling fraction for the large and small beams is fixed to 0.3 and 0.16. The wavelength of maximum absorption is plotted for the metafilm with just the large beam (white dashed line) and the metasurface with just the small-sized beams (black dashed line). The red dashed line indicates the location where *λ*=*P*. (**b**,**c**) Absorption map of a multiresonant metafilm with a fixed periodicity of 400 nm. (**b**) The impact of changing the size of just the small beams from 17 to 40 nm. (**c**) The impact of changing the large beams from 50 to 170 nm. (**d**,**e**) Experimental reflection spectra *R*_metasurface_ of six metafilms with wide and narrow beams interlaced as shown in the bottom panel of Fig. 3e. The spectra in **d** are taken from three metafilms for which the wide beams are fixed to a width of 120 nm and the small beams are 26, 32 and 38 nm. The spectra in **e** are taken from three metafilms composed of narrow beams with a fixed width of 26 and wider beams whose widths are 80, 100 and 120 nm. The vertical dashed lines indicate the locations of the tunable reflection dips.

As a next step, we show how the spectral absorption properties of the multiresonant metafilm can be manipulated by changing the geometry of one of the constituent beams. Figure 4a shows the simulated absorption map for the multiresonant metafilm under TM polarization. The period in this map is defined as the distance between the large-sized building blocks. The filling fraction for the large beams is fixed to 0.30 and small-sized building blocks is fixed to 0.16, amounting to a total *f*_Ge_=0.46. The white/black dashed lines indicate the location of the absorption peaks of the large/small-sized individual beams. The red line again shows the location at which *λ*=*P*. The two strong absorption features at long/short wavelengths can be attributed to the wide/narrow beams. Each of these resonances can individually be tuned by controlling the width of one of the beams at a deep-subwavelength scale while maintaining the 400 nm period. Figure 4b-4c show the impact of varying either the width of the smaller beams from 17 to 40 nm (Fig. 4b) or the width of larger beams from 50 to 170 nm (Fig. 4c). An increase in one of the beam sizes leads to a red-shift of the corresponding absorption resonance while leaving the other resonance unaffected.

Six metafilms with interlaced (wide and narrow) nanobeams were fabricated to experimentally verify the possibility to control the spectral absorption in a wavelength range of interest by controlling the geometry of the beam that resonates in the targeted wavelength range. For three of the metafilms, the wide beam size was kept constant at 120 nm and the width of the small beams was different for each film, 26, 32 and 38 nm, respectively. Figure 4d shows that the reflection spectra for this sample exhibits two resonant dips that can be attributed to optical resonances in the wide and narrow beams. A change in the narrow beam width from 26 to 38 nm shifts the short-wavelength resonance feature in the reflection spectrum from 560 to 640 nm while leaving the long-wavelength reflection dip at 865 nm unaffected. Figure 4e shows the opposite case where reflection spectra were taken from metafilms that all feature narrow beams with the same width of 26 nm, but wide beams with distinct widths of 80, 100 and 120 nm. The vertical dashed lines indicate the locations of the tunable reflection dips. Figure 4e shows that in this case the reflection dip on the long-wavelength side of the spectrum can selectively be red-shifted from 780 to 865 nm by increasing the width of the wider beams from 80 to 120 nm. The data agree well with the theoretically predicted shift as seen in Fig. 4b,c. Also, it shows that a high degree of control over the absorption spectrum can be attained with knowledge of the spectral absorption properties of the individual beams. The data in Fig. 4d, in particular, show that with this approach very strong absorption (>80%) can be achieved over a large bandwidth (>300 nm).

## Discussion

In summary, we have demonstrated how the absorption spectrum of an ultrathin metafilm absorber can be created by design. The resonant absorption properties of individual beams can effectively be encoded into the absorption properties of the metafilm. Following this approach, near-unity absorption was obtained in deep-subwavelength (∼50 nm) metafilms at various target wavelengths. Very strong absorption was also achieved over a large bandwidth by creating multiresonant metafilms. This opens opportunities to tailor the absorption spectrum for specific applications, such as solar energy harvesting and photodetection. The proposed concepts are very general and can be applied to unpolarized light by extending from one-dimensional to two-dimensional patterns (see Supplementary Fig. 3). More resonators with distinct resonant properties could also be added in the same plane or in multilayer metafilms to further enhance absorption. As such, this work provides new design strategies for planar optoelectronic devices, which traditionally have been limited by the intrinsic absorption properties of materials.

## Methods

### Metafilm fabrication

The Ge metafilms are prepared by standard thin-film deposition and electron-beam lithography. Electron-beam resist (2% PMMA) is spincoated on 150 nm thickness of Au-coated silicon substrate and a-Ge nanobeam array is produced by means of a subsequent lift-off process. The refractive index of a-Ge and the topography of the metafilm are characterized by means of ellipsometry and atomic force microscopy. Spectral reflectivity measurements were made using an optical microscope (Nikon C1). A broadband halogen lamp was used to illuminate the sample and the reflected light intensity was measured with a charge-coupled device camera (Acton Pixis1024 Princeton Instrument) connected with a monochromator (Acton SP2300i Princeton Instrument). The transmission response of the imaging systems was calibrated using an optically thick Au film deposited adjacent to the metafilms on the same substrate, which served as a reference mirror.

### Optical simulations

Full-field simulations are performed using the finite-difference time-domain technique. For the single-sized nanobeams, the height was fixed to 48 nm and the width of building blocks is varied as the periodicity changes (fixed Ge filling fraction of 0.3). For the multiresonant metasurface, the height of large building blocks is fixed to 48 nm and that of small ones is fixed to 30 nm, which matches with the height measured by atomic force microscopy. The reflection at each wavelength is calculated by monitoring the reflected power flow in the far-field region and the absorption in the metafilm region is calculated by Ohmic absorption of a-Ge, that is, *ω*·Im(*ɛ*) × |*E*|^2^, where *ɛ* is the complex dielectric constant of a-Ge obtained from ellipsometry. Power flow lines are plotted by calculating the Poynting vector of total field at steady state, that is, *S*_tot_=*E*_tot_ × *H*_tot_ (ref. 44).

## Additional information

**How to cite this article:** Kim, S. J. *et al*. Creating semiconductor metafilms with designer absorption spectra. *Nat. Commun.* 6:7591 doi: 10.1038/ncomms8591 (2015).

## Supplementary Material

Supplementary Information Supplementary Figures 1-3.
