# Supplementary material for: Creating semiconductor metafilms with designer absorption spectra
Source: Nat Commun. 2015 Jul 17;6:7591. doi: 10.1038/ncomms8591 (PMC4518292; doi:10.1038/ncomms8591)
Supplement: Supplementary Information — Supplementary Figures 1-3. [file ncomms8591-s1.pdf]

Supplementary figures

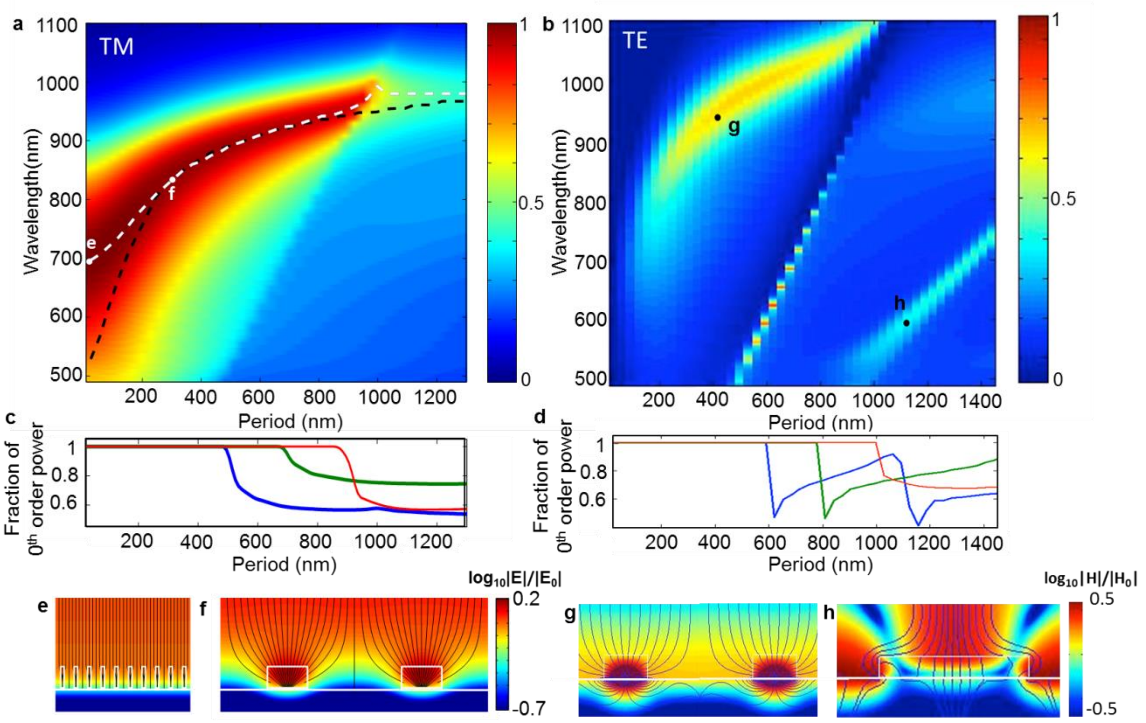

Supplementary Figure 1. Comparison of the absorption maps of a metafilm under TM and TE illumination. a. Simulated absorption map for Ge metafilms with different nanobeam periods *P* and widths *w* at a constant filling fraction of $f_{Ge} = 0.3$ (repeated from Fig.2a for easy comparison to the TE-illumination case). **b**. Absorption map for the same beam array as in panel a, but now under TE illumination. It can be seen that for this polarization the absorption is less than 70% for most of the wavelength-period combinations. Grating coupling under TE illumination is more significant in terms of enhancing absorption in the Ge as compared to that of TM illumination. **c,d** The fraction of power in the zeroth-order reflected beam is plotted at the illumination wavelengths of 600 nm (blue), 800 nm (green) and 1000 nm (red) for TM-illumination (c) and TE-illumination (d). **e,f.** The field distributions and overlaid powerflow for points e and f indicated in the absorption map for TM illumination. **g,h**. The field distributions and overlaid powerflow for points g and h indicated in the absorption map for TE illumination. The magnitude of the field is normalized to the incident field. The relatively strong absorption in region (g) is due to the excitation of an oscillating surface plasmon polariton along the metal/Ge interface and confined to the core of Ge nanobeam. Region (h) also shows the enhanced absorption due to the effects of a plasmonic resonance in which surface plasmon polariton travel back and forth between neighboring beams. These two effects are shown by the images of magnetic field distribution.

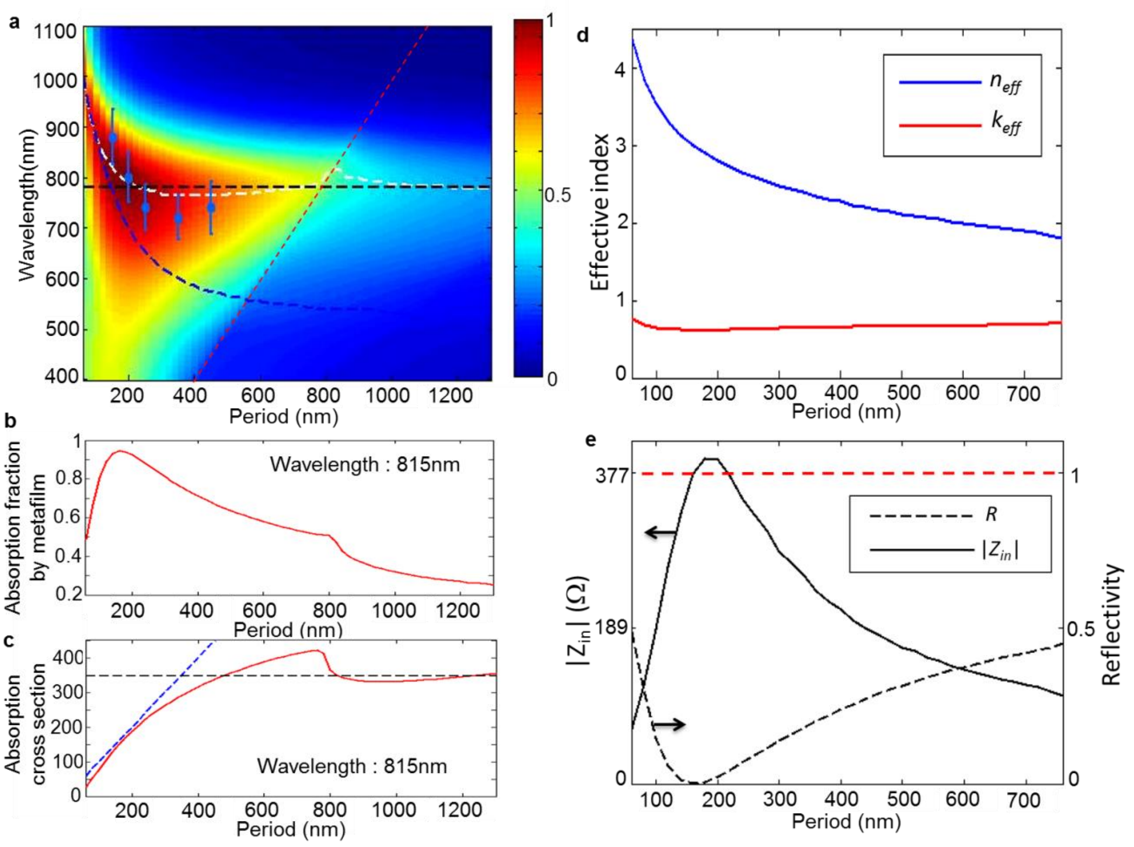

Supplementary Figure 2. Absorption map for a metafilm constructed from Ge nanobeams of a fixed width. a. Absorption map for a metafilm constructed from 60-nm-width Ge nanobeams**.** Whereas Fig. 2 of the main text showed an absorption map in which the filling fraction of Ge was kept the same for each array period, this absorption map shows the behavior for the case where the beam width was kept the same (at 60 nm) for each period. This choice implies that the filling fraction of Ge increases with decreasing period. The red dashed line again shows the condition $\lambda = P$ for reference. Similar to Fig. 2, the maximum absorption point at each period is indicated by a dashed white line. For sufficiently large period (> 200 nm), the dashed white line closely tracks the black dashed line, which indicates the simulated peak absorption wavelength of single 60-nm-width beams. Below the periodicity of around 200nm, the white dashed line diverges from the black dashed line and approaches the blue dashed line. The blue line indicates the condition of Fabry-Perot resonance location for the metafilm predicted by the first-order effective medium theory. Here, the beams are so close that the resonant behavior seen in the individual beams disappears due to the optical interaction between the beams. **b.** Plot of the absorbed fraction by metafilm versus the nanobeam period. The strongest absorption occurs for periods in the range from between 100 nm to 250 nm and reaches a maximum at around 200 nm. These periods are deep subwavelength and no higher diffracted orders are generated in reflection. The blue dots indicate the spectral location of the peaks in the experimental absorption spectra. The error bars show the bandwidth of absorption (FWHM/4). **c.** The absorption cross section of each building block in the array is shown (red line). For the period at which the absorption is at a

maximum, the cross section is almost equal to the period (blue line) and the absorption cross sections of the neighboring nanobeams cover almost the entire surface of the metafilm. At shorter periods, the beams lose their resonant behavior and the absorption cross sections reduce compared to those of the individual beams (black dashed line). For longer periods, the absorption cross section stays roughly constant and as a result ‘gaps’ appear between the absorption cross sections of neighboring beams with increasing period. In both cases this leads to a reduced absorption by the metafilm. This viewpoint is consistent with the viewpoint that the effective optical properties of the metafilm for the optimum periods are such that a good impedance match is achieved. **d.** Effective refractive index of a metafim with resonant building blocks at the wavelength λ = 815 nm where critical coupling to the metafilm (i.e. the strongest) absorption is achieved. **e.** Input impedance (Z_{in}) of metafilm on Au substrate (solid line) and reflectivity (dashed line) at the wavelength λ = 815 nm. At the optimal period of the nanobeam-array of 160 nm, the magnitude of the input impedance of $Z_{in} = 375 – j0.02\Omega$ closely matches the impedance of free space $Z_0 = 377\Omega$. For this reason, a critical coupling condition can be achieved at which we find that $((Z_{in} - Z_0) / (Z_{in} + Z_0) \approx 10^{-3})$. The input impedance (ratio of E & H) is calculated based on the wave interference inside the metafilm on the Au substrate ($Z_{in} = Z_{meta} \times (Z_{Au} + jZ_{meta}\tan(\beta_{meta}d_{meta})) / (Z_{meta} + jZ_{Au}\tan(\beta_{meta}d_{\mathrm{meta}}))$).

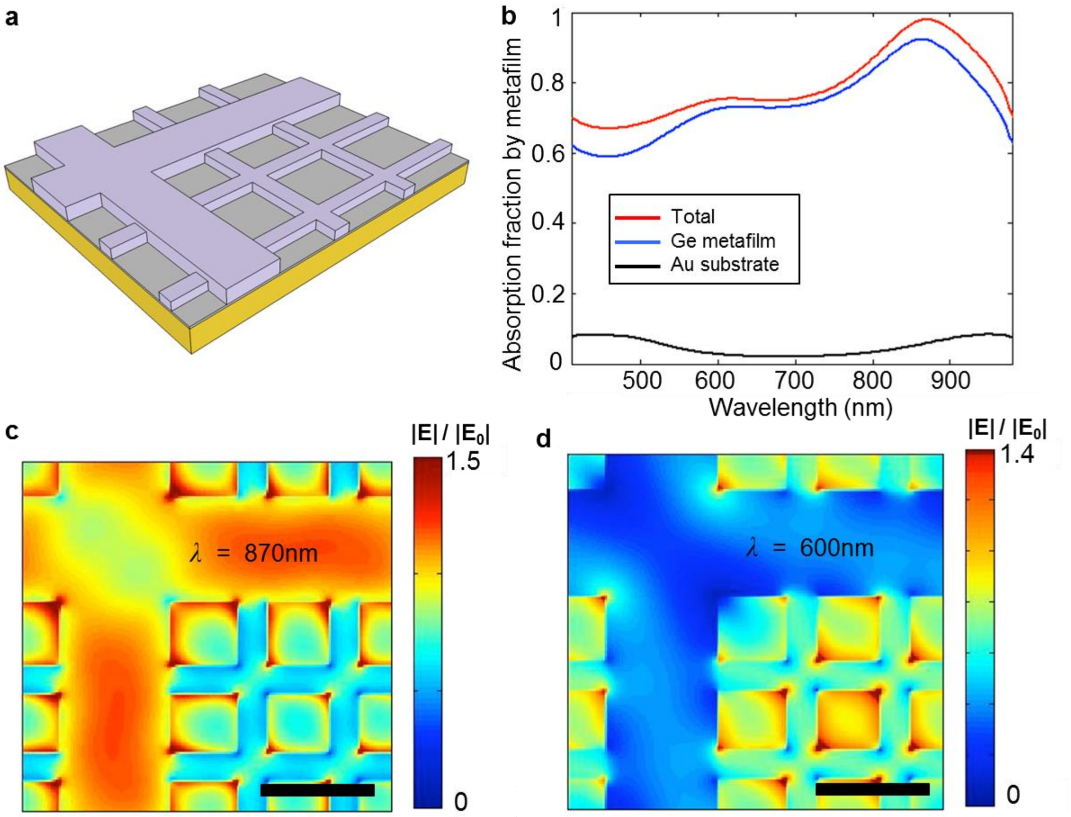

Supplementary Figure 3. Multi-resonant metafilm with a 2-dimensionl pattern that affords strong, broadband absorption of unpolarized light. a. Schematic of the a-Ge pattern/Au substrate structure featuring a pattern of crossed beam with both narrow (32-nm-width) and wide (120-nm-width) beams. This array was inspired by the multiresonant metafilm constructed from the linear beam-array and uses the same width beams. **b.** Plot of the fraction of spectral absorption for unpolarized illumination. The red line indicates the total absorption by the Ge metafilm and underlying substrate. The blue and black lines indicate the absorption of just the Ge metafilm and the Au substrate respectively. Here 1 corresponds to the case where all (100%) of the incident photons are absorbed. The graph clearly shows that very strong absorption in the Ge can be attained over a large bandwidth. **c-d.** Electric field distribution of the cross-sectional plane of the metasurface at the wavelength of 870 nm (c) and at 600 nm The magnitude of the field is normalized to the incident field (d). The plots indicate that the electric field is selectively enhanced in different spatial locations. The field localized preferentially in the larger beams when they are driven on resonance at long wavelength or in the small beams when they are driven on resonance at shorter wavelengths. Scale bar in panels c and d are 120nm.
